# Factors limiting presence: Perceptions of nurses working in a public psychiatric hospital

**DOI:** 10.4102/curationis.v45i1.2377

**Published:** 2022-11-28

**Authors:** Precious S. Motshabi, Emmerentia du Plessis, Francois Watson

**Affiliations:** 1 Job Shimankana Tabane Hospital, Rustenburg, South Africa; 2 NuMIQ Research Focus Area, Faculty of Health Sciences, North-West University, Potchefstroom, South Africa

**Keywords:** patients, perception, problem-solving, psychiatric nursing, quality improvement

## Abstract

**Background:**

Presence is a therapeutic skill that has a healing effect not only on the mental healthcare user but also the nurse. There was a need to explore nurses’ perceptions on factors that limit presence, especially in a public psychiatric hospital in a rural province such as North West, South Africa, where there are limited resources and nurses need to rely heavily on their therapeutic use of self.

**Objectives:**

To report on nurses’ perceptions of factors limiting presence when working in a public psychiatric hospital.

**Method:**

A qualitative descriptive inquiry was applied, with purposive sampling. Semistructured individual interviews were held with 10 nurses. Thematic data analysis was applied.

**Results:**

Intrapersonal factors that were found to limit presence arose from the view that mental healthcare users (MHUs) are difficult to engage with; the tendency to view ‘good care’ primarily as physical care with limited insight into presence was also recognised. Interpersonal and transpersonal factors related to difficulties in communicating with MHUs and in the work environment.

**Conclusion:**

Addressing factors that limit presence were found to require courage, the overcoming of interpersonal distance and a transformational process.

**Contribution:**

This article contributes important insights that can be used by nurse leaders to promote the practice of presence to improve the quality of psychiatric nursing care in developing contexts, such as a rural province in South Africa.

## Introduction

Taking into consideration that the practice of presence is a healing experience for both the mental healthcare user (MHU) and the nurse – characterised by fully and holistically being there for the other, attentiveness, comfort, doing for and competent performance of nursing procedures (Mohammadipour et al. 2017:4313) – it is worthwhile to reflect on the practice’s value. Presence is the nurses’ intention to reach out to MHUs, to be available to them and to attend to their needs – resulting in ‘good care’ (Timmerman, Baart & Vosman 2019:573). Such care is experienced by both the MHU and the nurse in meeting the MHU’s needs. This form of care has at least four dimensions, namely it is provided competently, in a relational way, so that the nurse is open to feedback and so that the care is given with an attitude of commitment (Du Plessis & Beurskens 2021:20).

Presence is the practice in which nurses align themselves with the patient in an attentive and dedicated way, learn to see what is important for the patient and come to understand what can be done and who they can be for the patient (Baart & Timmerman 2021:96). Presence is especially helpful and needed for patients who are difficult to reach, such as MHUs who have complex healthcare problems and who have difficulty in communicating their needs (Beurskens, Van der Linde & Baart 2019:31). This practice allows for and even invites talking about challenges such as mental health problems from a position of compassion and vulnerability, as opposed to seeing people who are vulnerable as being too difficult, a burden, too complex, or seeing vulnerability as unwanted and unworthy (Beurskens et al. 2019:31). When nurses practise presence, MHUs are seen and they experience that they are not being left alone; such experience continues if MHUs relapse, are difficult or seem to be different or deviating (Den Bakker & Willemse 2018:1). Furthermore, presence involves MHUs receiving the attentive, high-quality care and support that best fits them. The benefits of practising presence for nurses encompass good professional practice that is involved, dignified and conveys practical wisdom (Beurskens et al. 2019:31). This promotes joy in work and collaboration, as well as the experience of fulfilment and meaning in work (Kostovich 2012:167).

However, the practice of presence in the context of psychiatric or mental healthcare is limited by various factors. Nurses practising in mental healthcare institutions in South Africa face many challenges, such as unsafe working environments and staff shortages; these can be barriers to the provision of quality healthcare (Mulaudzi et al. 2020:a2081). Additionally, nurses working in mental healthcare institutions experience moderate to high levels of burnout and job dissatisfaction, leading to reduced empathy and quality of care (Payne et al. 2020:a1454). The focus of mental healthcare is typically on symptom management and discharge, with only limited emphasis on the nurse–patient relationship as a therapeutic tool (Sobekwa & Arunachallam 2015:1). While MHUs are vulnerable and mostly dependent on poorly resourced public mental healthcare services (Sobekwa & Arunachallam 2015:1), they can be perceived as dangerous and difficult to communicate with, leading to distancing and difficulties in nurse–patient relationships (Muhorakeye & Biracyaza 2021:1). Although quality of healthcare is emphasised and nurses are expected to have caring attitudes, the lack of resources, poor infrastructure, dissatisfaction and stigmatisation (Molefe & Sehularo 2015:472) could limit the practice of presence.

While research in the international and South African contexts confirms that presence leads to positive outcomes in mental healthcare for both the MHU and the nurse (Caldwell, Doyle & Morris 2005:853, Kalimashe 2020:1), there is also evidence of factors limiting the practice of presence (Esmaeili, Cheraghi & Salsali 2014:1). Such factors include misaligned understanding of teamwork, individual barriers resulting from a lack of motivation or holistic view, as well as organisational barriers involving a difficult work environment, lack of support and nursing staff shortages (Esmaeili et al. 2014:1). A further challenge relates to the difficulty of defining presence concerning when and how it is given, received or experienced – since this is not always consciously known to nurses, and the concept is often vaguely defined (Crane-Okada 2012:156). In the South African context, such as in the North West province, public psychiatric hospitals are criticised for being poorly managed and having inadequate resources that lead to mental healthcare that is degrading for recipients and poor job dissatisfaction for nurses (Wilson 2018); this, in turn, can lead to a limited practice of presence.

Thus, while it is important to enhance the practice of presence because of its healing and transformational effect on MHUs, it is necessary to first explore and describe the views of nurses working in the context of a public psychiatric hospital on factors that limit presence. This would enable the formulation of appropriate recommendations to improve the practice of presence. Therefore, the following research question guided the research: what do nurses working in a public psychiatric hospital in the North West province of South Africa perceive as factors that limit presence? The purpose of this study was to explore and describe the perceptions of nurses working in a public psychiatric hospital in the North West province of South Africa regarding factors that limit presence.

## Research methods and design

### Study design

A descriptive inquiry was used because a rich and straightforward description of the participants’ perceptions was needed (Sandelowski 2000:334). This design enabled the researcher to discover perceptions as they naturally existed among the participants. In reporting on this research, the authors adhered to the Consolidated Criteria for Reporting Qualitative Research (COREQ) checklist (Tong, Sainsbury & Craig 2007:349).

### Setting

The setting where the study took place was a tertiary public psychiatric hospital, one of two tertiary psychiatric hospitals in the North West province. The study hospital was chosen as it is situated in a rural area with limited resources, emphasising the need to rely on therapeutic interpersonal skills such as presence. The hospital has a capacity of 259 beds.

### Study population and sampling strategy

The study population comprised nurses who were rendering care to acute and chronic MHUs in a public psychiatric hospital in the North West province of South Africa. A total of 58 nurses formed the target population.

Purposive sampling was used to select participants to ensure a maximum variety of perceptions; this allowed for an exploration of both common and unique themes in the sample (Sandelowski 2000:334). Inclusion criteria were that nurses had to: have worked at the psychiatric hospital for more than one year, have provided acute and long-term inpatient care and be willing to sign informed consent forms to participate in the study. They further had to agree to audio-recording of their interview. Nursing students and newly employed nurses were excluded. Sampling and recruitment were carried out with the help of an independent person who was an employee at the psychiatric hospital. The independent person had no power relationship with the participants. She distributed recruitment material to potential participants and invited them to an information session held by the researcher. During the session, the independent person was also present, and she circulated a list so that potential participants could indicate whether they would be interested to participate; she also distributed the informed consent documentation. Potential participants were contacted by the independent person in the following week to arrange for an individual information session and to obtain informed consent. Participants were informed that their participation was voluntarily and that they could withdraw from the study at any time without reprisal.

The sample size was determined by data saturation (Saunders et al. 2017:1893). Based on the judgement of the researcher, research supervisors and the co-coder, data saturation was reached after conducting 10 interviews.

### Data collection

Data collection was done through individual semistructured interviews, supported by field notes, and took place between May and September 2019. Interviews were conducted by the first author as part of her master’s degree studies. She received training to conduct the interviews, and as a psychiatric nurse, she was experienced in communication skills. The participants’ perceptions were expressed through their words, gestures and facial expressions (Polit & Beck 2021:55). A semistructured interview schedule with open-ended questions guided the interview.

The interview schedule was developed based on the researcher’s understanding of the key concepts of the research, as informed by the context of the study, relevant literature and dictionaries. The researcher determined the key concepts to involve understanding *nurses’ perceptions of factors limiting presence.* In this study, *nurses* working at a public psychiatric hospital included all categories of nurses, being enrolled auxiliary nurses, staff (enrolled) nurses and professional nurses who were registered with the South African Nursing Council. *Perceptions* were defined for this study as ‘views’, ‘understanding’ and ‘opinions’ (Oxford University Press 2019a). The word ‘factor’ was defined as a ‘circumstance, fact, or influence that contributes to a result or outcome’ (Oxford University Press 2019b). ‘Limit’ in this research meant ‘restriction’ or ‘hindrance’ of something (Oxford University Press 2019c).

‘Presence’ was defined as the nurse’s intention to reach out to MHUs to be available and to attend to their needs through knowing the MHUs and knowing their unique needs with the intention of providing good care (Caldwell et al. 2005:853; Covington 2005:169; Duis-Nittsche 2002:20; McDonough-Means, Kreitzer & Bell 2004:25; Timmerman et al. 2019:96). Presence is interpersonal, intrapersonal and transpersonal in nature, which means presence happens inside the person and also occurs between two people, and it can be transferred between the two, and both the patient and the nurse benefit (McDonough-Means et al. 2004:25). Therefore, the key concepts associated with *nurses’ perceptions of factors limiting presence* were understood to have an *intrapersonal* dimension, namely the perceptions of nurses of MHUs and of their own need to provide good care; and an *interpersonal* and *transpersonal* dimension, namely the perceptions of nurses on factors that limit their ‘knowing of’ the MHUs, knowing their needs and providing good care (Table 1).

**TABLE 1:** Key concepts.

Key concepts	Description
Nurses	Professional, nurses, enrolled nurses, auxiliary nurses
	Working at a public psychiatric hospital in the North West province
Perceptions	Views
	Understanding
	Opinion
Factors	Circumstances
	Facts
	Influences
Limit	Restrict
	Hinder
Presence	Intra-, inter- and transpersonal dimension
	Reach out
	Be available
	Attend to needs
	Knowing the psychiatric patient
	Knowing the needs of psychiatric patients
	Good care

*Source*: Motshabi, P.S., 2020, ‘Perceptions of nurses at a public mental healthcare establishment in the North West Province on factors limiting presence’, MNS mini-dissertation, North-West University, Potchefstroom

Questions 1 and 5 of the interview schedule (Table 2) were formulated to gain insight into the intrapersonal dimension of presence, namely the participants’ views of MHUs and of their own needs as nurses to provide good care. Questions 2, 3 and 4 were formulated to explore interpersonal and transpersonal aspects, namely participants’ views, understanding and opinions of circumstances, facts and influences that limit, restrict and hinder knowing the MHUs, knowing their needs and providing good care. The semistructured interview schedule was structured in this way to allow the interview to flow from more general to more personal questions and was reviewed by the research team, and a trial run interview was held with a nonparticipant who met the inclusion criteria. The interview schedule was found to be adequate and relevant.

**TABLE 2:** Semistructured interview schedule.

Questions	Question focus
1. In your view, how do you describe the psychiatric patients admitted to your ward?	Intrapersonal dimension
2. What are factors that hinder you to get to know psychiatric patients?	Interpersonal and transpersonal aspects
3. What is limiting to you when you try to understand the needs of psychiatric patients?	Interpersonal and transpersonal aspects
4. What would you say are factors that are hindering you to provide good care to psychiatric patients?	Interpersonal and transpersonal aspects
5. What are your needs as a nurse to provide good care to psychiatric patients?	Intrapersonal dimension

*Source:* Motshabi, P.S., 2020, ‘Perceptions of nurses at a public mental healthcare establishment in the North West Province on factors limiting presence’, *MNS mini-dissertation*, North-West University, Potchefstroom

The interview schedule was applied flexibly, and communication techniques were used to build rapport with the participants so that the researcher could probe, clarify and actively listen to participants. The individual semistructured interviews were conducted in English. The researcher took descriptive, reflective, personal and demographic field notes after the interviews (Polit & Beck 2021:55). The individual semistructured interviews took place during work hours in one of the consulting rooms of the public psychiatric hospital and refreshments were served. Privacy, access and comfort were thus ensured. The interviews were scheduled at times that accommodated the workload and routines of the participants. Only the researcher (first author) and the participant were present during the interviews, which each lasted between 30 min and 45 min.

### Data analysis

Qualitative thematic analysis was applied (Sandelowski 2000:334). An experienced co-coder was requested to conduct independent co-coding to verify the identified themes and coding. Transcripts of the audio-recorded interviews and field notes were provided to the co-coder for data analysis. Transcripts were read carefully and meaningful segments were identified. Codes were developed from this data and used as a template to analyse the remaining data. As the researcher and co-coder independently analysed each new transcript, the codes were refined and modified. Consensus was reached on the codes, themes and subthemes that emerged from the data. These themes and subthemes form a rich descriptive summary of the perceptions of the participants.

### Trustworthiness

To ensure the trustworthiness of the data analysis, the researcher applied the four criteria outlined by Lincoln and Guba (1985:289): truth value, applicability, consistency and neutrality. Truth value was ensured by using the strategy of credibility, namely: prolonged engagement with the data; peer debriefing after each interview and during data analysis; and use of data saturation. To promote applicability and consistency, the researcher ensured transferability and dependability by providing a thick description of the research process, the context and research findings. Neutrality was ensured through confirmability via reflexivity; the thick description of the research also contributed to neutrality. Reflexivity, in turn, was achieved through personal reflection and peer debriefing among the researchers about research decisions. Reflective meetings and the involvement of a co-coder during data analysis also contributed to achieving reflexivity.

### Ethical considerations

Permission to conduct this research study was obtained from the Health Research Ethics Committee of the Faculty of Health Sciences of North-West University (reference number NWU-00074-18-A1). Permission was obtained from the North West Department of Health and from the management of the psychiatric hospital where the research took place. This approach ensured that the psychiatric hospital’s management and the participants were involved as collaborative partners. Voluntary, written informed consent was obtained from participants after a recruitment process through which fair selection was ensured. All research team members signed confidentiality agreements. The social value of this research is that the results, conclusions and recommendations add to the body of knowledge in psychiatric nursing and provide information on factors that hinder nurses working in a public psychiatric hospital to practise presence, in order that such factors can be addressed.

## Results

### Demographic information

The demographic information of the sample (Table 3) is as follows: participants’ ages ranged between 28 and 51 years old; eight professional nurses and two auxiliary nurses participated, and their work experience varied between 1 and 14 years. The initial intention was to include enrolled nurses also, but at the time of the study, no enrolled nurses were available to participate. The gender profile was that mostly men (*n* = 8) participated.

**TABLE 3:** Participants’ demographic information.

Demographic information	Participants (*n* = 10)
Age range (years)	28–51
Women	2
Men	8
Professional nurses	8
Auxiliary nurses	2
Work experience range (years)	1–14

*Source:* Motshabi, P.S., 2020, ‘Perceptions of nurses at a public mental healthcare establishment in the North West Province on factors limiting presence’, *MNS mini-dissertation*, North-West University, Potchefstroom

### Themes

Three themes and five related subthemes emerged, as indicated in Table 4. The ‘X’ symbol in the table indicates in which interviews the relevant themes emerged, as an indication that data saturation was achieved.

**TABLE 4:** Themes and subthemes with an indication of data saturation.

Theme	Subtheme	Participant									
		1	2	3	4	5	6	7	8	9	10
1. Mental healthcare users are difficult to engage with		X	X	X	X	X	X	X	X	X	X
2. Perceptions of psychiatric nurses on knowing the mental healthcare user and their needs	The psychiatric nurse obtains information about the mental healthcare user from different sources	X	-	X	X	X	X	X	X	-	-
	Perceptions of factors preventing nurses from knowing mental healthcare users	X	X	X	X	X	X	X	X	X	X
	Perceptions on factors impeding nurses from knowing the needs of mental healthcare users	X	X	X	X	X	X	X	X	X	X
3. Providing good care to psychiatric patients	Perceptions on factors preventing the nurse from providing good care	X	X	X	X	X	X	X	X	X	X
	Perceptions on what nurses need to provide good care to mental healthcare users	-	X	X	X	X	X	X	X	X	X

*Source:* Motshabi, P.S., 2020, ‘Perceptions of nurses at a public mental healthcare establishment in the North West Province on factors limiting presence’, *MNS mini-dissertation*, North-West University, Potchefstroom

#### Theme 1: Mental healthcare users are difficult to engage with

This theme relates to the intrapersonal dimension of presence, namely the participants’ view of MHUs. Participants perceived MHUs as being difficult to engage with because they demonstrate aggressive, impulsive, uncooperative and manipulative behaviours and have limited understanding. Further, the participants perceived MHUs as being dangerous, and the participants felt unsafe around them. They maintained that MHUs need constant supervision. One of the participants felt they are expected to advocate for the MHUs, and when they are not appreciated for this advocacy role but rather blamed by the MHU and their families, their practice of presence is limited.

The following quotes support this finding:

‘They can be naughty, aggressive. I mean aggressive patient with aggressive behaviour as we know mental healthcare users may experience hallucinations, delusions and they can be aggressive without knowing that they are aggressive towards a person.’ (Participant 1, auxiliary nurse, 1 year work experience)

‘Psychiatric patient they want twenty-four-seven observation. All the time because sometime you must make sure you are observing them, they are like small children from school, those that are school you have to say do this, do this always.’ (Participant 2, auxiliary nurse, 1 year work experience)

‘And remember I am the one who is supposed to be the advocate of the patient. If you are not appreciated, that also will put down the morale and then it limits the presence.’ (Participant 5, professional nurse, 13 years work experience)

#### Theme 2: Perceptions of psychiatric nurses on knowing the mental healthcare users and their needs

Three subthemes emerged. These themes relate to the interpersonal and transpersonal aspects of presence, specifically factors that limit knowing the MHUs and their needs.

**The psychiatric nurse obtains information about the mental healthcare users from different sources:** Participants interpreted this question to be related to the assessment of the MHUs. They shared that they obtained information mainly from consulting patient files, having discussions with family members and members of the multidisciplinary team. To a lesser extent, they asked the MHUs themselves.

The following quotes support this finding:

‘Collateral information from the family.’ (Participant 4, professional nurse, 3 years work experience)

‘Proper history from the person and maybe they are unable to, you know, to articulate or to tell their problem, or alternatively their relatives.’ (Participant 8, professional nurse, 4 years work experience)

**Perceptions of factors preventing nurses from knowing mental healthcare users:** Participants felt that MHUs withhold information or provide incorrect information. Language barriers, inadequate history taking and a lack of time to provide individual attention were also seen as limiting factors.

The following quotes illustrate this finding:

‘The patient is not open enough, so she does not want to disclose.’ (Participant 1, auxiliary nurse, 1 years work experience)

‘Because you will not reach all of them, you will not be able to interview all the patients that are there to know them.’ (Participant 4, professional nurse, 3 years work experience)

**Perceptions on factors impeding nurses from knowing the needs of mental healthcare users:** Participants perceived shortages of staff and communication difficulties as major limitations. Participants elaborated that staff shortages made it challenging to know MHUs’ needs. Frequent re-admissions take up much of the nurses’ time and limit nurses’ interaction with patients in the ward.

‘When patients are not able to talk their problems, we wouldn’t know about their needs are because patients won’t tell or say anything.’ (Participant 1, auxiliary nurse, 1 years work experience)

‘Because sometimes, they can be in the situation that is not right. Then by the time that you see that he is right after that then you call and ask him what is happening, and he will tell you this and that is happening according to the questions that you that you be asking him. Because you can’t talk to somebody when she/he is confused, they don’t listen to you.’ (Participant 2, auxiliary nurse, 1 years work experience)

‘Even the shortage of staff can make us not being able to know all the patients. Er, because you will not reach all of them, you will not be able to interview all the patients that are there to know them.’ (Participant 4, professional nurse, 3 years work experience)

#### Theme 3: Providing good care to mental healthcare users

A third theme was identified, and two subthemes emerged. The first subtheme resembles specific interpersonal and transpersonal factors limiting presence, namely factors limiting the provision of good care. The second subtheme implied specific intrapersonal factors that limit presence, namely perceptions on what nurses need to provide good care to MHUs.

**Perceptions on factors preventing the nurse from providing good care:** Participants perceived that ‘good care’ is care that is provided round the clock and that provides for the basic needs of MHUs. However, poor infrastructure and limited resources were reported to limit the nurses in their ability to provide for the basic needs of MHUs. This made the participants feel that they are not providing good physical care to the MHUs. In this regard, they experienced that members of the management team did not provide adequate support and they consequently felt demotivated and, as a result, demonstrated a relatively low level of performance.

‘Good care also it involves like for example, we have been trying for a very long time to be assisted teeth brushes, razors to cut their hair when we are sitting for grooming, we need all these things like during weekend we make them beautiful and then cut their hair and so on and so on, they are not there.’ (Participant 3, professional nurse, 14 years work experience)

‘We are giving care, we are taking care of mentally, physically, and psychiatrically maybe there is a shortage, there is no hot water.’ (Participant 4, professional nurse, 3 years work experience)

‘You know there is one thing I realised, we got managers who stay in the offices, they don’t know what is happening in the wards. They just stay in the offices, they don’t even know if you can ask him/her about the patients that are admitted in the wards, he/she cannot tell you, they don’t know anything because they just stay in the office.’ (Participant 9, professional nurse, 7 years work experience)

**Perceptions on what nurses need to provide good care to mental healthcare users:** Participants perceived a safe environment, protective clothing and adequate security as their most important needs to provide good care. They further expressed the need for a good remuneration package and substantial support from management. They viewed their physical health and mental strengths as imperative in providing good care. They further expressed the need for debriefing and counselling, as well as the need for empowerment through training.

The description is supported by the following quotes:

‘Provide clothes, provide hot water to the patients, give us protective clothing here. We work with our clothes, when I get here I have to take this shirt off and wear something that can protect me here in the hospital, nothing you don’t get it nowhere. … The security is not enough.’ (Participant 9, professional nurse, 7 years work experience)

‘Mm, me my needs is to further my studies so that I can know how to handle these psych patients.’ (Participant 2, auxiliary nurse, 1 years work experience)

‘We need the recognition; we need to be recognised in terms of the skills in terms of you as a human being.’ (Participant 5, professional nurse, 13 years work experience)

‘I think good managers laugh, one that encourages. … I think that (when the managers) see you doing something that is good they don’t encourage you to keep on doing it, because they keep quiet.’ (Participant 10, professional nurse, 3 years work experience)

## Discussion

### Summary of the key findings

The results describe the perceptions of nurses working at a public psychiatric hospital, revealing factors limiting presence. Three themes with five related subthemes emerged. The first theme represents a particular intrapersonal factor that limits presence, namely the perceptions of participants of MHUs as being difficult to engage with. The second theme with three subthemes revealed specific interpersonal and transpersonal factors that limit presence, namely factors limiting knowing the MHUs and their needs. The final theme with two subthemes is about good care and also contains interpersonal and transpersonal, as well as intrapersonal, factors that limit presence. Specific interpersonal and transpersonal factors limiting presence include the view that good care entails mostly basic, physical care. It also entailed views on factors that limited them to provide good care, namely limited infrastructure and poor resources and a lack of support from management. Perceptions on what nurses needed to be able to provide good care to MHUs pointed towards intrapersonal factors that limit presence and included the need for safety and security, a solid remuneration package, support and training.

### Discussion of the key findings

The discussion of the key findings follows the structure of intrapersonal, interpersonal and transpersonal factors that limit presence.

From the findings, it can be concluded that an intrapersonal factor that limits presence is the perception of MHUs as being difficult to engage with. Vincze, Fredriksson and Wiklund Gustin (2015:149) also found in Sweden that MHUs are viewed and experienced by nurses as aggressive and volatile. Such views can affect the care provided (Timmerman et al. 2019:96). According to Stevenson, Jack and LeGris (2015:2), nurses’ ability to carry out their professional nursing roles is indeed affected by the aggressive behaviour of patients, as was found in their Canadian study. Nurses find this situation stressful as they are torn between their desire to maintain a caring relationship with the patient and the need to diminish or eliminate the risk of violence towards themselves and others (Vincze et al. 2015:150). These circumstances require enduring courage from nurses to continue conveying their therapeutic presence with MHUs (Vincze et al. 2015:150).

Another possible intrapersonal factor that limits presence is view of the participants that good care mostly entails physical care, the availability of infrastructure and physical resources. The participants did not emphasise a connection with and a deep understanding of MHUs’ experiences as part of ‘good care’. As explained in Peplau’s nursing theory (Petriprin 2016), providing for the basic needs of the MHU is indeed part of the care provided by the nurse, and the frustrations of providing such care when resources are limited are recognised (WHO 2015). However, nursing theorists such as Peplau emphasised that ‘good care’ also entails *how* nurses apply themselves as an available resource; for example, this includes listening actively to the concerns of the patient to identify the needs of the patient (Petriprin 2016). Presence, as a way of providing good care, requires the ability and willingness of nurses to fully appreciate and enter, through relationship, the inner world of the MHU (Beurskens et al. 2019:31). As supported by more recent descriptions based on research conducted in the Netherlands (Baart & Timmerman 2021:90), and in the context of psychiatric or mental health nursing in the United States of America, ‘presence’ is described as knowing the uniqueness of individual patients, listening attentively with intense focus on the patient, engaging several potential channels for change, caring with confidence, creativity and perceived respect, involving the patient optimally and encountering mutually defined effective change (Caldwell et al. 2005:853).

Furthermore, interpersonal and transpersonal factors that limit the presence of caregivers relate to difficulties in communicating with MHUs and difficulties in the work environment. Literature confirms that communication barriers exist in mental healthcare (Gudde, Olso & Whittington 2015:449). However, Beurskens et al. (2019:33) observed that caregivers such as nurses – despite the unpredictable behaviour of MHUs and difficult work circumstances – make a concerted effort to connect with and attune to MHUs. The latter experience being seen as human beings, have the feeling of being trusted and consoled and possess a sense of hope. In previous research on this topic, Caldwell et al. (2005:853) conveyed that nurses providing nursing care to MHUs can create unique communication channels when the MHUs’ ability to communicate is limited. The importance of listening intently to identify unspoken wishes and to gain insight into the uniqueness of the MHU through building relationships is undeniably important (Caldwell et al. 2005:854).

Regarding the work environment, it is evident that the study participants highlighted the need for external help from management and additional resources to improve care; they further emphasised that management was not focused on building the relevant skills of carers. South African and international literature confirm that the lack of resources, large amounts of work and frequent admissions in public psychiatric hospitals can indeed increase workload, burnout, frustration, anxiety and a sense of not being cared for (Sobekwa & Arunachallam 2015:1; WHO 2015). However, Kuis, Goossens and Van Dijke (2015:173) suggested, based on their research in the Netherlands, that the presence approach offers a solution, as presence opens possibilities for a healthy and safe work environment in which nurses are encouraged; further, this enables the development of skills that nurses can apply to improve the nurse–patient relationship despite limited resources. In this regard, Ramalisa, Du Plessis and Koen (2018:1) emphasised the importance of supporting and acknowledging the efforts of nurses to increase their motivation and improve their care for patients. Beurskens et al. (2019:33) discussed how by providing good care through presence, nurses should be prepared to take the risk of being emotionally vulnerable to enable connection. They should further be able to know themselves and others, overcome interpersonal distance, develop a reflective state of consciousness and engage in a transformational process to respectfully negotiate their own and others’ needs.

### Strengths and limitations

The strength of the study is that the results revealed intrapersonal, interpersonal and transpersonal factors that limit presence, contributing to the body of knowledge in this field of nursing. The article provides important insights that nurse leaders in resource-poor settings can use to address factors that limit presence.

This study is limited to the views of nurses working at a public psychiatric hospital in the North West province of South Africa. However, the comparison of the findings of this research with existing literature, the thick description of the research process and the resulting discussion and recommendations for nursing open opportunities for wider application.

### Recommendations

Nurses working in psychiatric hospitals need to be encouraged, supported and empowered to negotiate meeting their own intrapersonal needs while providing good care through presence. This is necessary for providing for the needs of MHUs and should be achieved through supportive communication and building a trusting relationship with them. The intrapersonal, interpersonal and transpersonal dimensions of presence should be acknowledged and used as a framework to prepare nurses to practise presence. In the intrapersonal dimension, nurses should be encouraged to use their strengths, such as advocating for MHUs and involving the patients’ relatives. They should be made aware of the value of a presence approach in providing good care to MHUs, as well as overcoming barriers in connecting with and attuning to the needs of MHUs.

Self-care and self-awareness programmes should be made available to nurses so that they can resolve their own inner needs, do introspection and reflect on their perception of ‘good care’ and MHUs as human beings. Regarding the interpersonal and transpersonal dimensions, training programmes on providing nursing care through presence should be offered, and nurses should be guided in the therapeutic use of the self and in building trusting relationships to connect with and attune to MHUs and gain an understanding of their needs. Nurses should be guided in using their strengths and presence to build relationships with their supervisors and managers so that management is aware of staff needs. Nurses should further learn to negotiate for their workplace-related needs to be met, including needs for safety and an improved working environment.

Nurses should be acknowledged for their efforts to do the best they can to provide nursing care in difficult work circumstances. This can be done through relational management, where management builds relationships with nurses and obtains an understanding of nurses’ perceptions and needs. This acknowledgement can also be given through initiatives such as team-building meetings and meetings to convey appreciation and acknowledgement. Management can support nurses to find meaning in their work despite their circumstances, to be resourceful in creating a better environment for themselves and to communicate with management effectively to make their needs known. Further research should focus on MHUs’ views and needs regarding presence and good care, as well as the perceptions of management teams at psychiatric hospitals on presence, good care and factors limiting presence.

## Conclusion

The purpose of this study was to explore and describe the perceptions of nurses working in a public psychiatric hospital in the North West province of South Africa regarding factors that limit presence. The results of the study shine a light on the intrapersonal, interpersonal and transpersonal factors that limit presence. Nurses’ perception of who MHUs are; a one-sided focus on physical care as ‘good care’; limited communication between the nurse and the MHU; the need for improved infrastructure and resources; a more supportive work environment; and the need for training, including training on presence, are factors that limit presence. These findings inform the direction that needs to be taken to eliminate the factors that limit presence, in order that the practice of presence can be enhanced. Presence has a healing effect and can improve the quality of care to MHUs.
